# Dialysis-related carpal tunnel syndrome in the past 40 years

**DOI:** 10.1007/s10157-021-02122-8

**Published:** 2021-08-20

**Authors:** Masaki Hatano, Izuru Kitajima, Seizo Yamamoto, Masaki Nakamura, Kazuya Isawa, Tatsuya Suwabe, Junichi Hoshino, Naoki Sawa, Yoshifumi Ubara

**Affiliations:** 1 Department of Orthopaedic Surgery, Toranomon Hospital, 1-3-1, Takatsu, Kawasaki, Kanagawa 212-0015 Japan; 2 Department of Nephrology, Toranomon Hospital, 1-3-1, Takatsu, Kawasaki, Kanagawa 212-0015 Japan; 3 Okinaka Memorial Institute for Medical Research, Toranomon Hospital, Minato City, Tokyo Japan

**Keywords:** Carpal tunnel syndrome, Long-term hemodialysis, β2 microglobulin, β2 microglobulin clearance, Dialysis-related amyloidosis

## Abstract

**Purpose and method:**

Patients on hemodialysis develop carpal tunnel syndrome (CTS) due to an accumulation of dialysis-related β2 microglobulin (β2m) amyloid (DRA). In Japan, dialysis technology has progressed remarkably in the past 40 years and has increased the time until patients require surgery for CTS. However, unclear is whether the time from the start of hemodialysis to the first surgery for CTS is associated with β2m clearance by the different hemodialysis techniques. Therefore, we retrospectively evaluated β2m clearance, serum β2m levels, and the change in the length of this period in patients across 4 periods according to the year that first surgery for CTS was performed: period 1, 1982–1989; period 2, 1990–1999; period 3, 2000–2009; and period 4, 2010–2019.

**Result:**

A total of 222 patients who met the selection criteria were included. Mean β2m clearance was −1.8 ± 16.7% in period 1, and improved to 65.4 ± 8.6% in period 3. Accordingly, the serum β2m value after hemodialysis decreased significantly. The time from the start of hemodialysis to the first surgery for CTS was 12.4 ± 2.9 years in period 1 but increased to 21.8 ± 6.3 years in period 3. In multivariable linear regression analysis, the significant factors contributing to β2m clearance were periods 2, 3, and 4. In particular, the relation between removal of β2m and the extension of the dialysis vintage in period 1 and 2 was remarkable compared with periods 3 and 4.

**Conclusion:**

Our findings indicate that improvement of β2m clearance via advances in dialysis technology might result in a significant extension in the time between starting HD and the first surgery for CTS.

## Introduction

Advances in hemodialysis (HD) techniques are extending the lives of patients with chronic renal failure [1]. In Japan, almost 20,000 patients are on HD for longer than 20 years [1, 2]. Long-term HD is well known to increase the prevalence of complications. One of the most harmful complications of HD is dialysis-related amyloidosis (DRA), which is due to the deposition of β2 microglobulin (β2m) and adversely affects activities of daily living and quality of life [3, 4]. A common complication of DRA is carpal tunnel syndrome (CTS), in which amyloid deposits compress the median nerve [5]. In 1985, Gejyo et al. reported that β2m-amyloid deposition was found in operative specimens from patients undergoing CTS who had been on HD for 8–14 years [6]. Thereafter, with advances in HD technology, the time between starting HD and undergoing the first surgery for CTS increased. However, the reasons for this increase remain unclear [7]. Therefore, we divided patients who underwent CTS surgery in the past 40 years into 4 groups of 10-year periods, depending on the year of surgery, and compared β2m clearance, serum β2m levels, and the change in the time from the start of HD to the first surgery for CTS between the 4 groups.

## Methods

The main complications of dialysis amyloidosis are carpal tunnel syndrome, cervical spondylosis, spinal canal stenosis, trigger finger, bone cyst of the femoral neck, etc. We examined the records of operations for amyloid complications in the patients included in this study. The surgery for CTS was found to be the first surgery for amyloid complications. The relationship between carpal tunnel syndrome and dialysis amyloid was investigated as follows.

### Indication for surgery for CTS

Surgery for CTS was indicated only if physical signs of median nerve dysfunction were present according to electrodiagnostic nerve conduction study criteria, i.e., a distal motor latency of more than 4.5 m/second and a sensory latency of more than 3.5 m/second, in cases where surgery was requested because of classical symptoms of CTS, including numbness, tingling, and occasional pain in the hand.

### Patients

In this retrospective, single-center cohort study, we examined records of all patients who received regular HD at Toranomon Hospital, Tokyo, Japan, and underwent the first surgery for CTS at the Department of Orthopedic Surgery, Toranomon Hospital, between December 1982 and December 2019.

Patients were assigned to one of the following 4 periods, depending on when their first CTS surgery was performed: period 1, 1982–1989; period 2, 1990–1999; period 3, 2000–2009; and period 4, 2010–2019.

The study was performed in accordance with the Declaration of Helsinki and its revisions and approved by the local research ethics board (approval number: 2127-B).

### Clinical and laboratory parameters

The following clinical data were collected from the medical records, including laboratory data at the first surgery for CTS: age at start of HD; time (years) from the start of HD until surgery for CTS; sex; primary disease responsible for end-stage renal failure requiring HD; β2m clearance; and serum β2m, serum total protein, and serum albumin before and after HD. β2m clearance, which indicates the removal efficiency of β2m was evaluated with the following formula: (β2m value before dialysis−β2m value after dialysis) / β2m value before dialysis × 100.

### History of the HD method (Fig. 1)

**Fig. 1:**
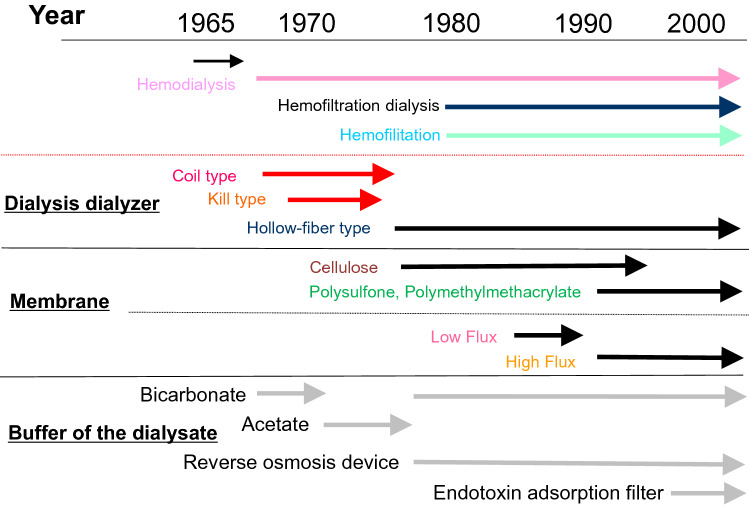
History of the dialysis method

Dialysis in Japan began in 1960, and by the year 2000, there had been major advances in dialysis technology, but the details of these advances have not been published in English paper, which can be found in Pub-Med service. Therefore, the history of dialysis was summarized as follows based on the documents left in our hospital and the books written in Japanese. In 1966, HD was introduced in our institute and was performed with a Kill dialyzer. This dialyzer was replaced in 1977 by a hollow fiber, low-flux dialyzer with a cellulose or cuprammonium cellulose membrane. A high-flux dialyzer with a high-performance membrane made of polysulfone, polymethylmethacrylate, or polyacrylonitrile was introduced in 1990; this dialyzer was better able to remove β2m, had better biocompatibility and caused lower complement activity. Because a large quantity of dialysate needed to pass through high-flux dialyzers, they required an ultrafiltration control system and a method to clean the dialysate. At first, an ion exchange resin and activated carbon were used to remove bacteria and chemical compounds from the dialysate. Subsequently, a reverse osmosis device was developed because the ion exchange resin used aluminum, and from about 1978 scientists became increasingly aware of the toxicity of aluminum. From about 2000, an endotoxin adsorption filter was used. This is the first time it has been used in Japan. At first, bicarbonate was used as the buffer for the dialysate. Acetate was introduced as the buffer in 1970, but bicarbonate was used again from 1974. The advances in dialyzer technology described above were accompanied after 1990 by marked improvements in the ability of HD to remove β2m.

There have been no major changes in hemodialysis technology since 2000, although on-line hemodiafiltration has been widely used in Japan since 2013 when it was covered by insurance.

### Statistical analyses

Data were summarized as percentages or as the mean ± SD. Categorical variables were analyzed with the chi-square test, and continuous variables, with the Kruskal–Wallis H test. The surgery probability in the 4 groups was calculated with the Kaplan–Meier method. Cox proportional hazards models were used to estimate hazard ratios (HRs) and 95% CIs for the association between the 4 groups and the period from the start of HD to the first surgery for CTS. Primary diseases causing HD were assigned to one of the following 3 groups: chronic glomerulonephritis, polycystic kidney, and other diseases. Multiple linear regression analysis was performed to evaluate the factors contributing to β2m clearance among selected demographic, biochemical, and clinical variables. Then, multiple linear regression analysis of the factors was carried out to identify those factors that were independently associated with β2m clearance and serum β2m levels after dialysis. The variance inflation factor (VIF) was used to check for multicollinearity in the model. All statistical analyses were performed with JMP PRO15 software. All p values were two-sided, and a *p* value < 0.05 was considered as indicative of statistical significance.

## Results

A total of 239 patients received regular HD and underwent the first surgery for CTS between December 1982 and December 2019. We excluded 17 patients because of a history of peritoneal dialysis and kidney transplantation, repeated surgery for CTS, lack of clinical data, or negative staining of Congo red for β2m in the surgical specimen. Therefore, 222 patients were eligible for inclusion in this study; 45.0% were men. The characteristics of patients are shown in Table 1.

**Table 1:** Baseline characteristics of study population

	All	Period 1	Period 2	Period 3	Period 4	*P*
		1982–1989	1990–1999	2000–2009	2010–2019	
Number of patients	222	14	39	81	88	
Man (%)	45.0	42.3	43.6	48.1	43.2	0.9193
Age at start of hemodialysis (years)	42.4 ± 12.1	45 ± 13.6	38.9 ± 11.6	41.0 ± 12.1	45.0 ± 11.3	**0.0138**
Age at the first surgery for CTS (years)	63.3 ± 9.3	57.4 ± 11.7	56.7 ± 9.2	63.1 ± 8.1	67.4 ± 7.7	**< 0.0001**
Time from the start of hemodialysis (years)	20.8 ± 6.7	12.4 ± 2.9	17.8 ± 5.4	21.8 ± 6.3	22.4 ± 6.7	**< 0.0001**
β2M clearance (%)	49.6 ± 28.2	– 1.8 ± 16.7	28.8 ± 22.3	65.4 ± 8.6	68.6 ± 3.9	**< 0.0001**
Serum β2M levels before dialysis (μg/L)	31.7 ± 9.2	42.6 ± 8.7	38.0 ± 5.6	28.2 ± 8.1	25.9 ± 3.9	**< 0.0001**
Serum β2M levels after dialysis (μg/L)	17.5 ± 14.0	43.2 ± 10.5	27.3 ± 9.8	9.9 ± 4.2	8.1 ± 1.7	**< 0.0001**
Total protain before dialysis (g/dL)	6.7 ± 0.6	6.9 ± 0.8	7.0 ± 0.3	6.7 ± 0.5	6.4 ± 0.6	**0.018**
Total protain after dialysis (g/dL)	7.3 ± 0.9	7.9 ± 0.5	7.3 ± 0.5	7.3 ± 1.0	6.9 ± 0.7	**0.0254**
Albumin before dialysis (g/dL)	3.3 ± 0.4	3.4 ± 0.5	3.4 ± 0.3	3.2 ± 0.3	3.2 ± 0.5	0.0548
Albumin after dialysis (g/dL)	3.4 ± 0.5	3.8 ± 0.5	3.4 ± 0.3	3.3 ± 0.5	3.3 ± 0.6	0.1219
*Primary diasese reqiring hemodialysis*						
Chronic glomerulonephritis	112	9	24	39	40	
Polycystic kidney disease	32	2	5	14	11	
Nephropathy of pregnancy/pregnancy toxemia	7	1	2	2	2	
Lupus nephritis	6			3	3	
Nephrosclerosis	4		1		3	
Diabetes	3		1		2	
Gouty kidney	3	1	1		1	
Obstructive urinary tract desease	3				3	
Chronic pyelonephritis	2			1	1	
Malignant hypertension	2				2	
Fanconi syndrome	1		1			
Acute glomerulonephritis	1			1		
Hypoplastic kidney	1			1		
Purpura nephritis	1			1		
Renal tuberculosis	1			1		
Amyloidosis	1				1	
Unknown	42	1	4	18	19	

In the 4 periods, mean β2m clearance was as follows: period 1,  – 1.8 ± 16.7%; period 2, 28.8 ± 22.3%; period 3, 65.4 ± 8.6%; and period 4, 68.6 ± 3.9%. Thus, β2m clearance improved across the 4 periods. As a result, serum β2m levels after dialysis decreased over the 4 periods, as follows: period 1, 43.2 ± 10.5 μg/L; period 2, 27.3 ± 9.8 μg/L; period 3, 9.9 ± 4.2 μg/L; and period 4, 8.1 ± 1.7 μg/L. Patients were significantly younger at age at the first surgery for CTS in periods 1 and 2 (57.4 ± 11.7 years and 56.7 ± 9.2 years, respectively) than in periods 3 and 4 (63.1 ± 8.1 years and 67.4 ± 7.7 years, respectively). The time from the start of HD until the first surgery for CTS increased across the 4 periods: period 1, 12.4 ± 2.9 years; period 2, 17.8 ± 5.4 years; period 3, 21.8 ± 6.3 years; and period 4, 22.4 ± 6.7 years.

Chronic glomerulonephritis was the most common primary disease responsible for end-stage renal failure, followed by polycystic kidney disease. Few patients had diabetes mellitus because they normally did not survive long enough to require surgery for CTS.

**Fig. 2:**
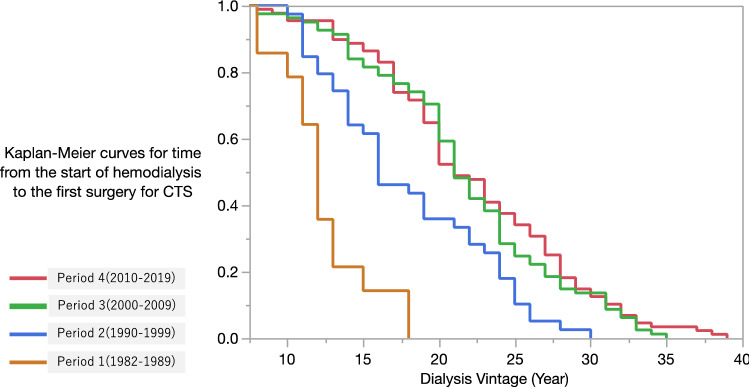
Kaplan–Meier estimates for the time from the start of hemodialysis to the first surgery for carpal tunnel syndrome by periods: period 1, 1982–1989; period 2, 1990–1999; period 3, 2000–2009; and period 4, 2010–2019 (*P* < 0.001 in the log-rank comparison between the curves)

Kaplan–Meier curves for the time from the start of HD to the first surgery for CTS showed significant differences between the 4 groups (*P* < 0.001, log-rank test) (Fig. 2).

In the adjusted analyses, the HR of time from the start of HD to the first surgery for CTS was lower in periods 2, 3, and 4 than in period 1 (Table 2; period 2 vs period 1: HR, 0.246; 95% CI 0.126–0.481; period 3 vs period 1: HR, 0.084; 95% CI 0.043–0.164; period 4 vs period 1: HR, 0.048; 95% CI 0.024–0.095). Age at start of HD was associated with the time between the start of HD and the first surgery for CTS (HR, 1.105; 95% CI 1.087–1.124).

**Table 2:** Association with increased risk of longer time from the start of hemodialysis to the first surgery for carpal tunnel syndrome in each of the 4 periods

Baseline factors		*n*	Hazard ratio	95% CI	*P*
Year					
	Period 1 (1982–1989)	14			
	Period 2 (1990–1999)	39	0.246	0.126–0.481	**<.0001**
	Period 3 (2000–2009)	81	0.084	0.043–0.164	**<.0001**
	Period 4 (2010–2019)	88	0.048	0.024–0.095	**<.0001**
Sex					
	Male	100	1.044	0.798–1.366	0.755
	Female	122	0.958		
Age at start of regular dialysis therapy (year)		222	1.105	1.087–1.124	**< .0001**
Primary disease					
	Chronic glomerulonephritis	112			
	Polycystic kidney	32	1.119	0.737–1.698	0.599
	Otherwise	78	1.158	0.861–1.559	0.322

The factors contributing to β2m clearance were evaluated by multivariable regression analysis (Table 3). Compared with period 1, periods 2, 3, and 4 were significantly associated with increased β2m clearance. The factors contributing to serum β2m after HD were also evaluated by multivariable regression analysis (Table 3). Period 3 and 4 were significantly associated with decreased serum β2m after dialysis and serum β2m levels before HD were significantly associated with increased serum β2m after HD.

**Table 3:** Multivariable regression analysis for β2m clearance and serum β2m levels after dialysis

Baseline factors		Estimate	Lower 95%	Upper 95%	β	*P*
*Multivariable regression analysis for β2M clearance*						
Year						
	Period 2	– 8.189	– 14.525	– 1.853	– 0.174	**0.0123**
	Period 3	22.859	17.431	28.286	0.565	**< .0001 **
	Period 4	26.249	18.486	34.013	0.59	**< .0001 **
Sex						
	Male	0.977	– 2.316	4.27	0.034	0.554
Primary disease						
	Chronic glomerulonephritis	1.84	– 3.137	6.818	0.059	0.461
	Polycystic kidney	– 0.674	– 8.144	6.796	– 0.014	0.857
Dialysis Vintage		– 0.046	– 0.812	0.721	– 0.12	0.905
Age at start of regular dialysis therapy (year)		– 0.359	– 0.755	0.037	– 0.157	0.07
Serum β2 microglobulin levels before dialysis (μg/L)		– 0.248	– 0.723	0.227	– 0.083	0.299
Total protain before dialysis		2.471	– 3.592	8.533	1.289	0.417
*Multivariable regression analysis for serum β2M levels after dialysis*						
Year						
	Period 2	1.659	– 0.97	4.289	0.07	0.211
	Period 3	– 8.881	– 11.134	– 6.628	– 0.436	**<.0001**
	Period 4	– 9.302	– 12.524	– 6.079	– 0.415	**<.0001**
Sex						
	Male	– 0.857	– 2.223	0.51	– 0.059	0.214
Primary disease						
	Chronic glomerulonephritis	– 0.045	– 2.11	2.021	– 0.003	0.966
	Polycystic kidney	– 0.787	– 3.888	2.314	– 0.033	0.613
Age at start of hemodialysis (year)		0.176	0.012	0.34	0.153	**0.036**
Serum β2 microglobulin levels before dialysis (μg/L)		0.606	0.408	0.803	0.401	**<.0001**
Total protain before dialysis		0.082	– 2.435	2.598	0.003	0.948

## Discussion

In this study, we examined whether developments in HD techniques over the past 40 years improved β2m clearance and serum β2m levels and affected the time between starting HD and undergoing the first surgery for CTS. Indeed, we found that β2m clearance improved from period 1, the first 10-year period, to period 4 and that serum β2m levels also decreased. The time from the start of HD to the first surgery for CTS increased from period 1 to period 4.

Gejyo et al. extracted β2m with a molecular weight of 11,000 Daltons from amyloid tissue of patients with CTS, and β2m-related amyloid subsequently became known as dialysis-related amyloidosis (DRA) [8]. Yamamoto et al. reported that age, duration of HD, type of dialysis membrane used, chronic inflammation, advanced glycation, oxidation, and direct cell toxicity were also major risk factors for DRA [9]. Küchle et al. compared 10 patients treated with HD with low-flux cuprophane membranes with 10 patients treated with HD with high-flux polysulfone membranes; after 6 years, they found no clinical signs of DRA in patients dialyzed with high-flux polysulfone membranes but CTS or osteoarticular lesions or both in 8/10 of patients dialyzed with cuprophane low-flux HD membranes. Furthermore, serum levels of β2m were significantly lower in patients dialyzed with high-flux polysulfone membranes [10].

Schwalbe et al. compared the prevalence of β2m amyloidosis in 1988 and 1996 and found that high-flux synthetic dialysis membranes were used more frequently in 1996, as was reverse osmosis water plus bicarbonate buffer for dialysate preparation. Furthermore, the prevalence and severity of β2m amyloidosis unexpectedly decreased from 1988 to 1996 [11].

Lornoy et al. reported that treatment with on-line hemodiafiltration with a highly permeable and biocompatible membrane is an efficient, well-tolerated, and safe technique that leads to a low prevalence of dialysis amyloidosis and a superior phosphate clearance [12].

Nakai et al. reported that in Japan the prevalence of CTS surgery decreased from 48.0% in a 1999 survey to 23.2% in a 2010 survey, and from 70.8% to 51.5% among patients with a dialysis vintage of 25 years or more [13]. In Poland, from 2005 to 2008 the duration of dialysis therapy in patients with CTS ranged from 4 to 30 years (mean, 16.05 years) [14]. In Germany, from 2009 to 2011 CTS due to amyloidosis was seen in as many as 22% of patients after 10 years of HD and in 50% of patients after 14 years of HD [15].

Hoshino et al. presented the results of a comparison of patients assessed in Japanese nationwide surveys in 1998 and 2010. The reduction of the time until the first surgery for CTS was most prominent in patients with a longer dialysis vintage, younger patients, and patients with lower pre-dialysis β2m levels [16]. Hoshino et al. reported that the odds ratios of the first surgery for CTS doubled with every 5-year increase in dialysis vintage and were highest for patients aged 60–70 years [17]. Nishi et al. reported that CTS appeared significantly earlier than trigger finger. In the advanced phase of DRA, knee joint pain was a major cause of decreased activities of daily living in patients with clinical DRA [18].

In Table 1, we showed that the number of patients underwent CTS surgery increase from the period 1 to the period 4. This is due to the fact that the number of dialysis patients in Japan has been increasing every 10 year; the number of patients in Japan who have been on dialysis for more than 10 years was 13,000 in 1985, 33,000 in 1995, 59,000 in 2005 and 89,000 in 2015 according to a survey by the Japanese Society for Dialysis Therapy Renal Data Registry Committee [19]. Hoshino et al. reported that the number of patients receiving the first surgery for CTS was 647 with dialysis vintage of 17.4 ± 6.0 years on 1998 cohort in Japan, and was 2157 with dialysis vintage of 18.2 ± 9.5 years for 2010 cohort [17].

In conclusion, dialysis technology in Japan has progressed remarkably in the past 40 years and has had a positive effect on the occurrence of CTS related to accumulation of DRA. β2m clearance improved across the 4 decades and serum β2m before and after HD consequently decreased. The time from the start of HD to the first surgery for CTS increased. In particular, the relation between removal of β2m and the extension of the dialysis vintage in period 1 and 2 was remarkable compared with period 3 and 4. From the above previous reports and our results, we believe that the better removal of β2m via the advancement of dialysis technology might contribute to the extension of the dialysis vintage of patients undergoing surgery for CTS.

## Limitations

The current study has several limitations. First, we used the first surgery for CTS as a proxy for advanced DRA, but information is needed on other complications of DRA, such as trigger finger and destructive spondyloarthritis. The surgery for CTS was found to be the first surgery for amyloid complications. The relationship between carpal tunnel syndrome and dialysis amyloid was investigated. Significant extension in the time between starting HD and the first surgery for CTS was explained by β2m clearance alone. Second, the frequency of the primary disease requiring HD was different from that in the Japanese dialysis therapy renal data registry [1], where polycystic kidney is the most common primary disease among all patients receiving HD.

## Key learning points

Long-term hemodialysis is well known to increase the prevalence of complications. A common complication of dialysis-related amyloidosis (DRA) is carpal tunnel syndrome (CTS). However, there are few reports investigating incidence for DRA and a historical perspective of the historical characteristics of DRA have not been clarified.

This study aimed to clarify the historical perspective on the characteristics of DRA and the involvement of β2 microglobulin according to the incidence of first-time carpal tunnel release surgery as proxy for DRA onset.

The time from the start of hemodialysis to the first surgery for CTS was 12.4 ± 2.9 years in 1980–1990 but increased to 21.8 ± 6.3 years in 2000–2010.

Our findings indicate that improvements in the efficiency of β2m removal by hemodialysis decreased the accumulation of DRA and, thus, increased the time until patients require their first surgery for CTS.

## Abbreviations

Alb: Albumin
CTS: Carpal tunnel syndrome
CI: Confidence interval
DRA: Dialysis-related amyloidosis
RDT: Regular dialysis therapy
TP: Total protein
β2m: β2 Microglobulin
